# Technological Advances in Molecular Diagnostic Methods for Hereditary Diseases in Preconception and Prenatal Settings

**DOI:** 10.3390/cimb48080756

**Published:** 2026-07-25

**Authors:** Deyuan Kong, Jianing Zhao, Haichang Diao, Shuyao Qiu, Yuanyuan Peng, Tingting Liu

**Affiliations:** Department of Epidemiology and Health Statistics, School of Public Health, Southeast University, Nanjing 210009, China; kongdeyuan0011@163.com (D.K.); 230259502@seu.edu.cn (J.Z.); haichang_0420@163.com (H.D.); qiushuyao321@163.com (S.Q.); yuanyuanpeng0119@163.com (Y.P.)

**Keywords:** carrier screening, preconception genetic testing, next-generation sequencing, noninvasive prenatal testing, cell-free DNA

## Abstract

Precision prevention and control of genetic diseases represent a major public health challenge. This paper provides a structured narrative review of advances in molecular diagnostic technologies across the preconception, preimplantation, and prenatal stages over the past five years. In the preconception phase, next-generation sequencing has become central to carrier screening, while long-read sequencing significantly enhances detection capabilities for complex variants. In the preimplantation phase, research has increasingly focused on non-invasive preimplantation genetic testing, leveraging maternal contamination quantification algorithms and deep learning models to address DNA contamination challenges. During the prenatal phase, stratified diagnostic strategies combining chromosomal microarray analysis and whole-exome sequencing have improved the diagnostic evaluation of fetal structural anomalies. Simultaneously, non-invasive prenatal testing is expanding to include microdeletion/duplication and monogenic disease screening, though positive screening results still require invasive diagnostic confirmation. Future trends lie in multi-technology integration, multi-omics data fusion, and artificial intelligence-assisted decision-making, aiming to enhance resolution while balancing health-economic considerations and ethical standards.

## 1. Introduction

Genetic disorders and congenital birth defects have emerged as significant global public health challenges. According to the World Health Organization, approximately 7.9 million children are born with serious birth defects annually, accounting for approximately 6% of births worldwide, many of which have a genetic origin [1]. However, traditional screening methods exhibit substantial technical limitations, which have catalyzed an urgent clinical demand for efficient and safe molecular diagnostic technologies.

Each reproductive stage presents distinct clinical questions. The preconception stage centers on carrier screening. Although next-generation sequencing (NGS)-based expanded carrier screening (ECS) enables simultaneous multi-gene detection, it remains constrained by residual risk and an insufficient capacity to detect complex genetic variants. During the preimplantation genetic testing (PGT) stage, limited biopsy sample volumes frequently lead to allelic dropout (ADO) and whole-genome amplification bias, while embryonic mosaicism significantly compromises diagnostic accuracy. Non-invasive preimplantation genetic testing (niPGT) analyzes embryo-derived cell-free DNA released into spent embryo culture medium before embryo transfer and should not be confused with celocentesis, an invasive prenatal sampling procedure performed after implantation. Although niPGT avoids direct embryo biopsy, its clinical application remains investigational because of low DNA input, embryonic mosaicism, and contamination from maternal or polar-body DNA [2,3,4,5]. In the prenatal phase, while invasive diagnostics offer high resolution, the associated risk of miscarriage remains a concern, and stratified clinical diagnostic strategies integrating chromosomal microarray analysis (CMA) and sequencing technologies are not yet fully optimized. Additionally, non-invasive prenatal testing (NIPT) is hindered by interference from confined placental mosaicism (CPM) and low fetal DNA fractions. Consequently, NIPT remains a screening rather than a diagnostic test, and positive results require invasive confirmation before irreversible clinical decisions; the choice of confirmatory procedure should account for the possibility of confined placental mosaicism.

This review synthesizes recent advances in molecular diagnostics across the preconception–PGT–prenatal continuum with an explicit focus on clinical utility. We provide stage-specific decision pathways outlining which measures should be used and when, thereby offering practical guidance for reproductive medicine.

## 2. Methods

We conducted a structured narrative review of molecular diagnostic technologies for hereditary diseases in preconception, preimplantation, and prenatal settings. PubMed and Web of Science were searched for English-language studies published from 1 January 2020 to 30 June 2026. Search terms covered carrier screening, preimplantation and prenatal genetic testing, CMA, ES, GS, LRS, OGM, NIPT, cfDNA, multi-omics, and artificial intelligence. Reference lists of relevant reviews, guidelines, consensus statements, and key studies were also screened, and earlier landmark studies were included when necessary.

High-quality evidence was defined as professional guidelines, systematic reviews, meta-analyses, prospective or multicenter cohort studies, and large retrospective studies with clearly defined populations, reference standards, and clinically relevant outcomes. Small case series and proof-of-concept studies were included only for emerging technologies with limited higher-level evidence. Studies lacking relevance, methodological detail, or analytical and clinical outcome data were excluded. Recommendations from ACMG, ACOG, ASRM, SMFM, and ISPD were used to assess clinical indications and implementation status. The corresponding guidelines, consensus statements, and reference citations are provided in Supplementary Table S1.

## 3. Results

We outline the general workflow for molecular diagnostics throughout the reproductive process. Initially, couples should be offered expanded carrier screening to identify those at high risk for recessive genetic disorders. Subsequently, couples identified as being at high risk may opt for PGT to select unaffected embryos or proceed with natural conception followed by early prenatal monitoring. During pregnancy, routine NIPT and serial ultrasound surveillance serve as the primary screening tools for common chromosomal abnormalities and major structural defects. In the event of a positive NIPT result, invasive diagnostic confirmation is mandatory before irreversible clinical decisions are made, with the choice between chorionic villus sampling and amniocentesis guided by the suspected abnormality and the likelihood of confined placental mosaicism. If fetal ultrasound anomalies are detected, CMA is generally recommended as a first-line diagnostic modality. In cases where CMA yields negative results but structural anomalies persist, ES or GS should be performed to identify potential causative single-gene variants.

### 3.1. Preconception Molecular Diagnostic Methods

#### 3.1.1. Carrier Screening for Genetic Conditions

**Figure 1:**
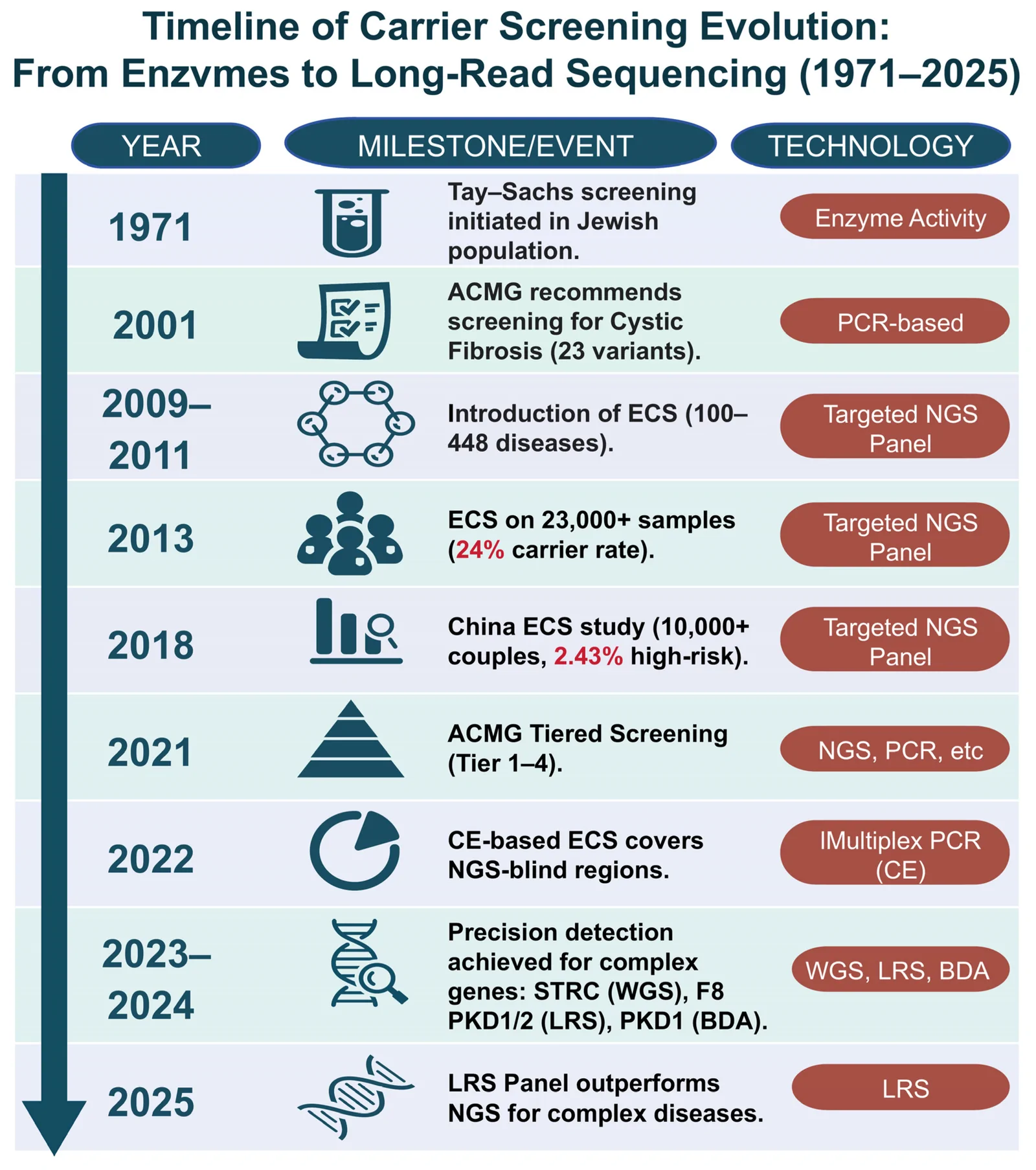
Timeline of the Evolution of Carrier Screening. Note: ECS, expanded carrier screening; NGS, next-generation sequencing; ACMG, American College of Medical Genetics and Genomics; CE-based, capillary electrophoresis-based; GS, Genome Sequencing; LRS, long-read sequencing; BDA, blocker displacement amplification. 1971: Carrier screening for Tay-Sachs disease was initiated within the Ashkenazi Jewish population using enzyme activity assays, pioneering the era of single-disorder carrier screening. 2004: The ACMG recommended screening for 23 pathogenic variants associated with cystic fibrosis (CF23) utilizing PCR-based targeted mutation detection. 2009–2011: ECS was first implemented, increasing the scope from single disorders to 100–448 recessive conditions through targeted NGS panels. 2013: An ECS study of over 23,000 samples for more than 400 conditions revealed that 24% of individuals were carriers for at least one disorder, utilizing NGS-based targeted gene panels. 2018: An ECS program targeting 11 recessive disorders via gene panels was conducted among more than 10,000 Chinese couples, identifying 2.43% as high-risk couples. 2021: ACMG proposed a tiered screening framework (Tier 1–Tier 4) using NGS and PCR-based methods, recommending screening for disorders with a carrier frequency of ≥1/200 in preconception and prenatal populations. 2022: Establishment of CE-based ECS methods. The application of capillary electrophoresis multiplex PCR screening technology enables the detection of genes that are challenging for NGS coverage, including heterozygous deletions in *SMN1* (Spinal Muscular Atrophy), *CGG* repeats in *FMR1* (Fragile X Syndrome), and large structural rearrangements in *CYP21A2* (Congenital Adrenal Hyperplasia). 2023–2024: High-precision detection of complex genes. Key advancements include: precise detection of STRC CNVs associated with deafness based on GS; accurate localization of F8 breakpoints in Hemophilia A using the LRS-based CAHEA method; comprehensive analysis of PKD1 and PKD2 for Autosomal Dominant Polycystic Kidney Disease via the LRS-based CAPKD method; and effective elimination of PKD1 pseudogene interference in ADPKD utilizing BDA technology. 2025: The screening utility of an LRS panel for screening five complex genetic disorders was evaluated, demonstrating superior performance compared to conventional NGS.

Carrier screening is defined as genetic testing performed in individuals who do not manifest the disorder but may carry a pathogenic variant associated with that disorder [6]. Currently, ECS can screen for over a thousand recessive single-gene disorders in a single test, focusing on autosomal recessive diseases and certain X-linked conditions (e.g., thalassemia, hereditary hearing loss). In recent years, third-generation sequencing (long-read sequencing) has demonstrated significant advantages (Figure 1). Despite these technological advancements, current clinical bottlenecks in carrier screening include not only technical blind spots but also the complexities associated with calculating “residual risk.”

Due to its high throughput and sensitivity, NGS has emerged as the core detection methodology for ECS. Its primary strategies in carrier screening are as follows:

Targeted gene panel sequencing is currently the most widely utilized strategy, operating via the probe-based enrichment of target regions prior to sequencing. An NGS-based panel covering 220 conditions in a Chinese population demonstrated that 62.3% of individuals harbored at least one pathogenic variant; however, this approach exhibits geographical limitations [7].

Exome sequencing focuses on the protein-coding regions of the genome and facilitates the identification of unexpected pathogenic variants, albeit at a higher cost. A retrospective cohort study revealed that at least one pathogenic or likely pathogenic variant was detected in 71.9% of subjects via exome sequencing, demonstrating its potential clinical utility [8].

Whole-genome sequencing encompasses both the coding and non-coding regions of the genome, yielding the most comprehensive genetic information. Nevertheless, its clinical application remains restricted by considerable costs and massive data volumes. Currently, whole-genome sequencing is recommended for universal newborn screening for hearing loss, with relevant studies demonstrating the accurate identification of copy-number variants (CNVs) in the *STRC* gene and its pseudogene, *STRCP1* [9].

Although ECS has greatly expanded the breadth of screening, detection strategies based on short-read NGS still face technical blind spots when encountering complex genomic regions.

Third-generation sequencing, also known as long-read sequencing (LRS), not only improves variant detection but also enables the characterization of complex variants, including cis–trans configurations and precise breakpoints [10,11]. In a recent carrier screening study involving five complex monogenic diseases (spinal muscular atrophy, α-/β-thalassemia, 21-hydroxylase deficiency, and fragile X syndrome), targeted LRS identified 236 carrier variants, whereas conventional NGS detected only 56.4% of these variants, highlighting the ability of LRS to resolve genomic regions affected by pseudogenes, repeats, and complex structural variation [12]. LRS is transitioning from research to clinical application, particularly for repeat expansions, complex or balanced structural variants, haplotype phasing, methylation abnormalities, and difficult genomic regions containing pseudogenes, segmental duplications, or high-GC sequences. In patients with rare diseases who remained undiagnosed after short-read sequencing, LRS provided an additional diagnostic yield of approximately 10%, highlighting its complementary value in unresolved cases [13]. A direct comparison involving 310 families with undiagnosed neurological disorders further demonstrated its advantages in resolving complex variants and enabling allele-specific interpretation [14]. Emerging frameworks integrating long-read sequencing, diploid genome assembly, pangenome references, and artificial intelligence (AI)-assisted interpretation may further reduce current diagnostic blind spots [15,16]. However, requirements for high-molecular-weight DNA, higher costs, limited throughput, and insufficiently standardized analytical pipelines currently restrict its use as a routine first-line test. In clinical practice, a tiered strategy based on phenotype and initial testing results is therefore appropriate, with LRS used as a complementary test after negative ES/GS, particularly when repeat expansions, complex structural variants, pseudogene-associated regions, or variant phasing are suspected.

Innovative molecular diagnostic technologies specifically designed for targeted diseases are continuously emerging. Technologies such as blocker displacement amplification (BDA) and capillary electrophoresis-based multiplex PCR screening can effectively address the detection gaps inherent in NGS.

#### 3.1.2. Preimplantation Genetic Testing

Preimplantation genetic testing involves the screening of genetically normal embryos for transfer following conventional in vitro fertilization (IVF) or intracytoplasmic sperm injection (ICSI), thereby preventing the transmission of hereditary diseases and improving live birth rates. PGT encompasses testing for aneuploidies (PGT-A), monogenic/single-gene defects (PGT-M), and chromosomal structural rearrangements (PGT-SR). While NGS constitutes the current mainstream platform, other technologies such as single-nucleotide polymorphism (SNP) arrays and Comparative Genomic Hybridization (CGH) remain in use. Several studies have demonstrated that low-depth whole-genome sequencing facilitates the integrated detection of PGT-A, PGT-M, and PGT-SR [17,18,19,20]. Notably, third-generation sequencing technologies (including nanopore sequencing and SMRT sequencing) demonstrate their unique value. Some third-generation sequencing strategies eliminate the need for a proband, significantly shortening turnaround times to within several hours [21,22,23,24,25]. Nevertheless, these findings warrant further validation through larger-scale cohorts.

For a long time, trophectoderm (TE) biopsy has been regarded as the “gold standard”; however, while it resolved the issue of poor representativeness associated with early polar body biopsy, the potential biological safety risks inherent to this invasive procedure remain a focal point of clinical concern.

#### 3.1.3. Investigational Non-Invasive Preimplantation Genetic Testing

niPGT refers to the analysis of embryo-derived cell-free DNA (cfDNA) in spent embryo culture medium during in vitro culture, before embryo transfer. It aims to avoid trophectoderm biopsy and is therefore non-invasive with respect to the embryo. However, its clinical use remains investigational because of variable concordance with trophectoderm or whole-embryo results, low DNA input, maternal or polar-body DNA contamination, embryonic mosaicism, and the absence of prospective evidence demonstrating improved implantation or live-birth outcomes. In contrast, celocentesis is an invasive prenatal procedure performed after implantation, usually in early pregnancy, in which extraembryonic coelomic fluid is obtained by needle aspiration for fetal genetic analysis. These approaches differ in developmental stage, sample source, indications, procedural risks, and clinical maturity; therefore, they should not be considered interchangeable.

**Figure 2:**
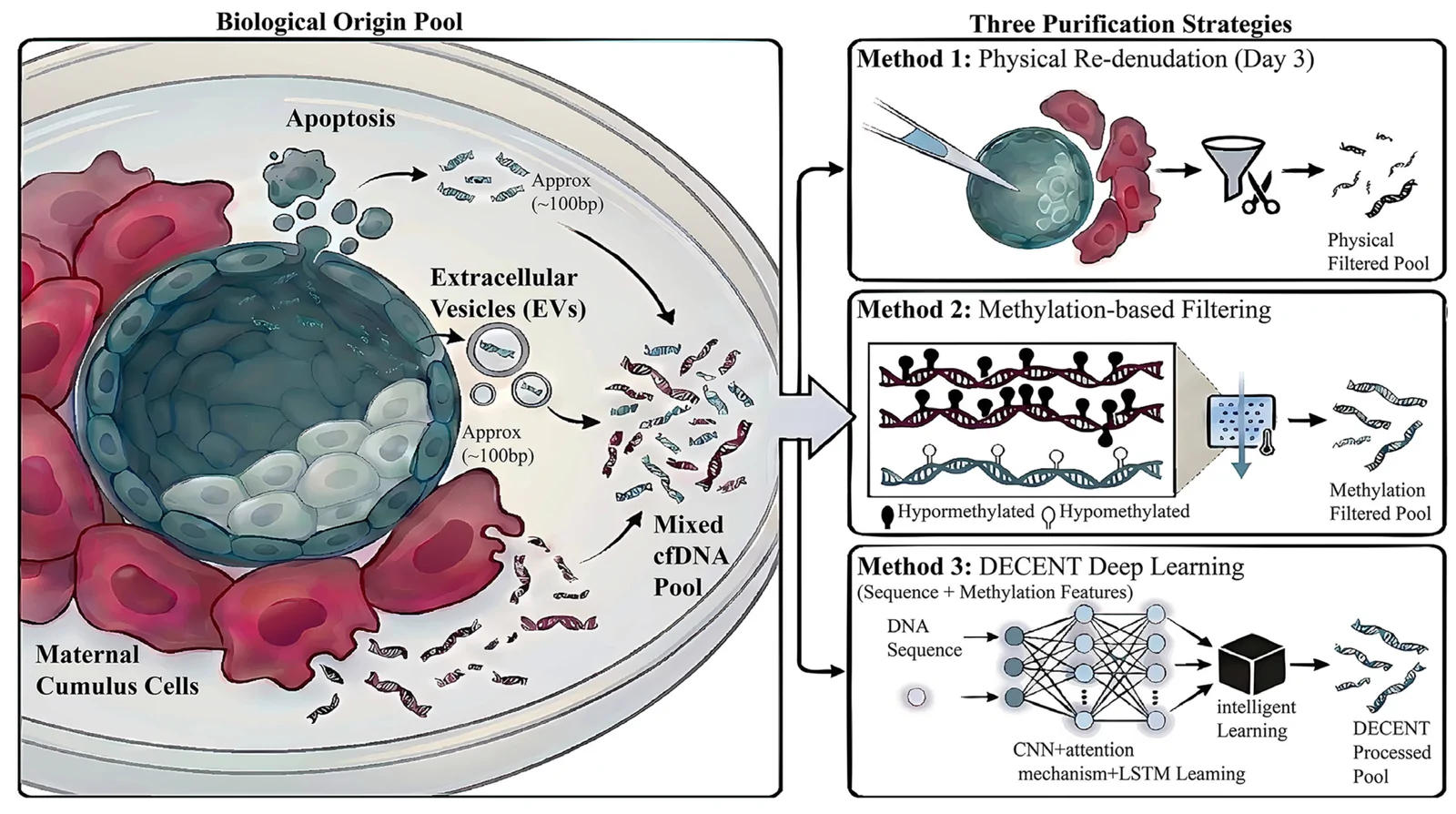
Origins of cfDNA in SECM and mechanistic comparison of three purification strategies for maternal DNA removal. Trophectoderm cells and the Inner Cell Mass (ICM), as core components of the blastocyst (green), are the primary contributors of embryo-derived cfDNA (cell-free DNA) in SECM. Maternal cell contamination essentially results from the interference of maternal cumulus cells (red) and other sources with low-concentration embryonic cfDNA; maternal cumulus cells are the principal source of maternal cell contamination in embryo culture medium. This figure illustrates three recent methods for maternal cell contamination removal: (1) Physical Re-denudation (Day 3): Mechanical removal of maternal cells reduces contamination interference. Nevertheless, its efficacy is limited and it cannot eliminate MCC originating from polar bodies. (2) Epigenetic Identification based on DNA Methylation: The core principle exploits methylation differences between embryonic and maternal cells, with blastocyst cfDNA exhibiting hypomethylation. This method relies on the annotation of methylated regions. (3) Deep Learning DECENT: This model integrates a Convolutional Neural Network (CNN) to extract local features of DNA methylation sequences, an Attention mechanism to capture multi-level features, and Long Short-Term Memory (LSTM) to capture long-term dependencies and temporal patterns. By simultaneously utilizing cfDNA sequence information and methylation status, it achieves precise classification of embryonic versus maternal reads. This approach requires no additional experiments but is dependent on training data and cannot address polar body contamination.

While conventional niPGT primarily targets embryos derived from intracytoplasmic sperm injection, recent research has expanded its application to the general in vitro fertilization population, demonstrating that the sensitivity and specificity of niPGT are comparable between IVF and ICSI embryos [26]. Furthermore, addressing the diagnostic challenge of chromosomal mosaicism, Li et al. utilized the whole blastocyst as the gold standard to eliminate sampling bias inherent in trophectoderm biopsies [27]. By proposing a 50% mosaicism threshold, they increased the overall concordance between spent culture medium-cfDNA and the whole blastocyst to 87.2% (*n* = 41 blastocysts), thereby enhancing the diagnostic accuracy of niPGT for mosaic embryos [27]. Additionally, the transcriptome of extracellular vesicles (EVs) in spent embryo culture medium (SECM) has emerged as a novel biomarker for assessing embryonic developmental potential. The identification of unique transcriptomic signatures in EVs from aneuploid embryos (e.g., *PPM1J*, *TMED10*) provides a non-DNA-based dimension for niPGT [28]. However, the clinical translation of niPGT faces a critical barrier: maternal DNA contamination. Recent strategies to overcome maternal cell contamination (MCC) include physical stripping, epigenetic tracking based on differential methylation, and sequence feature extraction utilizing deep learning, such as the DECENT model [2,3,4,5]. Although DECENT demonstrates the feasibility of computationally separating embryonic and maternal cfDNA [4], the available evidence is mainly derived from model-development datasets. Its generalizability across laboratories and clinical benefit therefore require prospective external validation [4]. Furthermore, computational maternal DNA decontamination may not fully resolve DNA contributions from polar bodies [2,3]. Figure 2 illustrates these methods for addressing maternal cell contamination, including physical re-denudation, methylation-based filtering, and the DECENT deep learning model.

Other technological innovations have also expanded the potential applications of PGT. Advanced techniques, such as PGT-Sean sequential analysis and Virtual Long Read, facilitate the mitigation of ADO and enable proband-independent detection; however, these methods require further validation through large-scale, multicenter studies [29,30]. Family-specific polygenic risk score models, combined with embryo whole-genome reconstruction, allow for effective risk stratification of polygenic disorders. For mitochondrial diseases, a “pronuclear transfer + PGT” stratified intervention strategy can be implemented, though its adoption remains constrained by ethical considerations and the availability of oocyte donation [31,32,33].

Furthermore, multi-omics and AI approaches are being explored to support embryo assessment. Extracellular-vesicle transcriptomics and RNA-based PGT may provide additional biological information regarding embryo developmental competence [28,34]. Preimplantation methylomic profiling and other epigenetic approaches may provide research insights into embryo development; however, their clinical utility remains unproven, and they should not currently be regarded as routine embryo-selection or diagnostic tools [35]. AI models based on static or time-lapse embryo imaging have shown potential for embryo quality assessment and ploidy prediction. However, most available studies are retrospective and rely on selected datasets or surrogate outcomes, and model performance may vary across clinics, imaging platforms, and patient populations. Prospective multicenter evidence demonstrating improvements in implantation or live-birth outcomes remains limited. Therefore, AI should currently be regarded as a decision-support tool rather than a replacement for embryologist assessment or genetic testing [36,37,38,39,40].

### 3.2. Prenatal Molecular Diagnostic Methods

Although invasive diagnostics (CMA/ES) provide the definitive genetic information, the associated risk of miscarriage limits their widespread application. NIPT is safer but remains a screening tool. Table 1 compares the clinical performance of major molecular diagnostic technologies in prenatal settings.

#### 3.2.1. Invasive Molecular Diagnostic Technologies

Invasive prenatal molecular diagnostics, which involve obtaining fetal or placental tissue samples via invasive procedures to determine fetal genetic status using molecular biological methods, remain the “gold standard” for prenatal diagnosis. Sampling modalities include chorionic villus sampling, amniocentesis, and percutaneous umbilical blood sampling. Beyond specific combined applications, invasive prenatal diagnosis generally adopts a tiered diagnostic strategy: CMA serves as the first-line test for structural anomalies; if CMA is negative despite persistent suspicion, ES, GS, Optical Genome Mapping (OGM), or other technologies are considered.

**CMA and ES: A Precise Prenatal Diagnostic System from Chromosomal to Single-Gene Levels.** Traditional chromosome analysis often fails to meet the clinical diagnostic requirements for subtle genetic abnormalities due to its limited resolution (detecting only anomalies > 5–10 Mb) and prolonged turnaround time (2–3 weeks) [45]. Current international guidelines recommend CMA as the first-line invasive diagnostic tool for fetuses with structural anomalies [46]. However, CMA is limited by its inability to detect balanced chromosomal rearrangements or single-gene mutations. ES serves as a critical complement to CMA, addressing the diagnostic gap for monogenic variants. A multicenter prospective cohort study demonstrated that ES identified diagnostic single-gene variants in 8.5% of 610 fetuses with structural anomalies [47]. Furthermore, the inability of ES to detect variants in non-coding regions may cause some pathogenic variants to be missed.

**Advantages of Emerging Prenatal Molecular Diagnostic Technologies.** Other molecular diagnostic technologies have demonstrated their advantages. Growing evidence supports parent–fetus trio GS as a potential first-tier test for fetal structural anomalies. Compared with the sequential strategy of CMA followed by ES, GS can simultaneously assess copy-number variants, single-nucleotide variants, small insertions and deletions, and selected structural and non-coding variants in a single assay. In a prospective study of 111 fetuses with structural or growth abnormalities, trio GS detected all 22 diagnoses identified by CMA followed by ES, while requiring less input DNA and reducing the mean turnaround time from 31 ± 8 days to 18 ± 6 days [43]. In smaller cohorts, GS also identified exon-level copy-number variants and structural variants that were missed by CMA and ES [48]. However, meta-analyses have not demonstrated a consistent overall diagnostic-yield advantage for GS, suggesting that its principal value lies in consolidating diagnostic workflows and broadening variant coverage rather than markedly increasing diagnostic yield [49]. GS may therefore be considered a first-tier diagnostic option for fetuses with major or multisystem structural anomalies in centers with established analytical and interpretive expertise. Nevertheless, owing to cost, interpretive complexity, incidental findings, incomplete detection of certain forms of mosaicism, repeat expansions, and methylation abnormalities, as well as the need for confirmatory testing, GS cannot yet fully replace CMA followed by ES [49,50].

Copy-number variation sequencing (CNV-seq), based on low-depth whole-genome sequencing, is primarily utilized for detecting chromosomal aneuploidies and CNVs. The integrated application of chromosome analysis, quantitative fluorescent PCR, CNV-seq, and ES substantially enhances variant detection rates, shortens diagnostic turnaround times, and facilitates the identification of complex cases [51,52,53,54,55]. Both CMA and CNV-seq detect aneuploidy and CNVs, but CNV-seq cannot detect uniparental disomy (UPD). Notably, CNV-seq can detect lower levels of mosaicism than CMA [56]. Clinical selection between CMA and CNV-seq should be based on sample type, suspected variant classes, and other diagnostic findings.

In the field of monogenic diseases, Comprehensive Analysis of Thalassemia by Single-molecule sequencing (CATSA), based on LRS, enables one-stop detection of α- and β-globin gene variants in high-risk pregnancies. This technique identifies rare variants and provides a 7.9% (15/191) incremental yield over traditional PCR-based methods, although it entails higher costs and longer turnaround times [57]. Additionally, OGM is an emerging cytogenomic technology based on the labeling of ultra-high-molecular-weight DNA and whole-genome imaging. It enables the detection of balanced and unbalanced structural variants and copy-number variants while preserving long-range chromosomal architecture. In a prospective cohort of 204 prenatal samples, OGM achieved a diagnostic yield of 25%, compared with 22.06% for CMA and 18.14% for karyotyping; combining OGM with karyotyping further increased the diagnostic yield to 29.41% [58]. Recent multicenter and prenatal studies have also demonstrated high concordance and reproducibility between OGM and conventional cytogenetic methods [59,60]. OGM complements genome sequencing by further characterizing balanced translocations, inversions, complex chromosomal rearrangements, and the orientation of duplicated segments. Combining OGM with long-read sequencing may provide greater diagnostic resolution than sequencing alone and facilitate the interpretation of complex structural variants [61]. However, OGM cannot detect single-nucleotide variants or small insertions and deletions and remains limited in certain centromeric and telomeric regions and in detecting low-level mosaicism. Therefore, OGM is best regarded as a complementary cytogenomic method rather than a replacement for sequencing technologies. Table 2 provides a technical overview of prenatal screening for various variant types.

#### 3.2.2. Non-Invasive Prenatal Testing

The cell-free DNA fraction analyzed by NIPT is predominantly released from placental trophoblasts and therefore represents a placental proxy for the fetal genome rather than a direct fetal sample. This biological origin explains discordant results caused by confined placental mosaicism and other placental abnormalities. In contemporary prenatal medicine, technologies based on placenta-derived cfDNA in maternal plasma have substantially advanced prenatal screening. Table 3 compares different non-invasive prenatal testing technologies.

The technological foundation of NIPT lies in low-depth whole-genome sequencing of maternal plasma cfDNA, primarily leveraging genome-wide NGS [72] to identify chromosomal copy number abnormalities through high-efficiency bioinformatic counting. Its initial success was based on its high screening performance for common fetal aneuploidies. A large-scale study demonstrated that its sensitivity for trisomies 21, 18, and 13 exceeds 98.9%, with a specificity greater than 99.9%, confirming NIPT as a primary frontline tool for screening common fetal aneuploidies in women of reproductive age [42,62,73,74]. With increasing sequencing depth, the scope of NIPT has expanded from basic aneuploidy screening to include sex chromosome aneuploidies and microdeletion/microduplication syndromes (often referred to as “NIPT-Plus”). However, its positive predictive value (PPV) varies significantly; the likelihood of a fetus being affected following a positive NIPT result is influenced by specificity, false-positive rates, and the background prevalence of specific conditions [75]. Because the prevalence of common fetal trisomies increases with maternal age, the same analytical sensitivity and specificity generally result in a lower PPV and a correspondingly higher proportion of false-positive results among younger or otherwise low-risk pregnant women [76,77]. In a study of 81,838 pregnancies, the combined PPV for common autosomal aneuploidies was significantly higher in the high-risk group, which included advanced maternal age, high-risk serum screening results, or abnormal ultrasound findings, than in the non-high-risk group (85.2% vs. 59.2%, *p* < 0.01) [76]. Another study involving 47,855 pregnancies found that the PPV for sex chromosome abnormalities was significantly lower in women aged <30 years than in those aged 30–34 years [78]. Therefore, maternal age, disease prevalence, ultrasound findings, previous screening results, and the specific chromosomal abnormality should be considered when interpreting a positive NIPT result, and invasive diagnostic confirmation is required before irreversible clinical decisions are made [76,77,78]. The choice between chorionic villus sampling and amniocentesis should consider gestational age, the suspected chromosomal abnormality, ultrasound findings, and the likelihood of confined placental mosaicism. Because both NIPT and chorionic villus sampling assess placental material, amniocentesis may be preferable when confined placental mosaicism is a major concern. When a fetal structural abnormality is detected by ultrasound, NIPT should not be used as a substitute for invasive diagnostic testing. These pregnancies should be offered diagnostic testing, generally including chromosomal microarray analysis, because NIPT does not comprehensively assess pathogenic copy-number variants, balanced rearrangements, mosaicism, or monogenic disorders.

**The clinical scope of NIPT is rapidly expanding from the detection of chromosomal abnormalities to include single-gene disease (SGD).** Relative Haplotype Dosage (RHDO) analysis exhibits exceptional accuracy, typically yielding results within 11 days with a failure rate of only 4% [64]. However, this approach has traditionally been proband-dependent and faces technical hurdles in consanguineous families. Emerging technologies, such as Relative Mutation Dosage (RMD) and Nanopore-based RHDO, now enable proband-independent NIPT [68,79]. Furthermore, targeted sequencing techniques—specifically Unique Molecular Identifiers (UMI) and Circulating Single-Molecule Amplification and Resequencing Technology (cSMART)—have demonstrated superior performance in SGD detection [69]. Recent prospective cohort studies indicate that Collaborative Allele-aware Targeted Enrichment Sequencing (COATE-seq) can precisely isolate fetal genomes from maternal plasma. This facilitates the synchronous detection of aneuploidies, microdeletions, and single-gene variants [63,66]. These studies mark a shift in NIPT from traditional chromosomal screening toward comprehensive testing for heterogeneous genetic disorders.

**Technical innovations have helped NIPT overcome bottlenecks.** NGS is constrained by its time-consuming nature, complexity, and high operational costs. Multiplex digital PCR has emerged as a rapid complementary approach for Trisomy 21 detection, achieving 100% sensitivity and specificity within a 2–3 h turnaround time [80]. The cfDNA component analyzed by NIPT is predominantly derived from placental trophoblasts, and its proportion in maternal plasma is conventionally referred to as the fetal fraction (FF). Low FF remains the primary driver of NIPT failures and false-negative results, with an FF of <4% being significantly associated with adverse pregnancy outcomes [81]. Although NIPT has a high negative predictive value for common autosomal trisomies, a negative result substantially reduces but does not eliminate the possibility of fetal chromosomal disease. False-negative results may arise from low placental DNA fraction, placental–fetal discordance, fetal or placental mosaicism, technical limitations, or abnormalities outside the validated detection scope. Negative predictive values should therefore be interpreted in relation to the tested condition, assay scope, fetal fraction, and pretest risk. Although techniques such as chromosomal phasing and FF amplification have been developed to enhance detection performance, these methods require further validation in large-scale cohorts [82,83]. Deep-learning models have shown preliminary feasibility for detecting low-abundance fetal variants [84,85]. However, their calibration, interpretability, cross-population generalizability, and prospective clinical utility remain insufficiently validated [84,85]. Genome-wide haplotype analysis facilitates comprehensive detection across the genome, but its clinical utility requires validation in larger studies [71]. Substantial evidence has now accumulated regarding the use of NIPT in multiple pregnancies, which have traditionally presented a substantial technical challenge. Large-scale prospective multicenter studies confirm that NIPT for Trisomy 21 in twin pregnancies yields sensitivity and specificity exceeding 99%, demonstrating performance comparable to that in singleton pregnancies; however, further evidence is required for its application in triplet or higher-order pregnancies [86,87,88].

**Breakthroughs in cell separation technologies and the integration of multi-omics data are providing novel perspectives for NIPT.** Specifically, single circulating trophoblast (SCT) testing has demonstrated high analytical performance by efficiently enriching extremely rare intact fetal cells from maternal blood, although its clinical translation requires broader evidence and the development of automated platforms [70,89]. Additionally, genome-wide bisulfite sequencing and other methylation-based approaches have shown proof-of-concept potential for placental or chromosomal assessment [90]. Methylation profiles vary with gestational age, placental cell type, and pregnancy complications, while reference datasets and evidence of clinical benefit remain limited [91,92]. These approaches should therefore remain investigational. More broadly, many emerging NIPT technologies remain in the research phase and require analytical standardization, external validation, and prospective clinical evaluation before routine implementation.

## 4. Discussion

This review delineates the evolution of molecular diagnostic technologies across the preconception, preimplantation, and prenatal stages over the past five years. However, the rapid evolution of technologies has concomitantly engendered novel challenges, including data fragmentation, controversies regarding health economics, and the blurring of ethical boundaries.

Clinical counseling must underscore the core concept that “a negative screen does not equal zero risk.” Although ECS covers hundreds of conditions, NGS-based testing still carries “residual risk.” This stems primarily from technical limitations (e.g., difficulty covering deep intronic variants or complex structural variants) and biases in estimating population carrier rates when calculating residual risk. More importantly, the generation of preconception ES/GS data should not be used solely for a single reproductive decision but should facilitate the construction of a Family Genetic Health Record. Extending genomic data from the preconception stage to the prenatal and even neonatal periods can significantly enhance diagnostic efficiency. For instance, parental carrier status determined via preconception screening can serve as critical background data for subsequent NIPT interpretation, effectively resolving the issue of missing information in haplotype phasing. This full-cycle perspective facilitates more coherent reproductive health management.

Modern genomic diagnosis increasingly relies on the integration of advanced sequencing technologies with synergistic clinical–research workflows. As patient phenotypes evolve over time and additional clinical information becomes available, systematic reanalysis can identify diagnoses that were missed during the initial evaluation [93]. In prenatal diagnosis, multidisciplinary review and genotype-driven reverse phenotyping can refine fetal phenotypes, improve variant interpretation, and provide additional diagnoses after the initial analysis [94,95]. However, the diagnostic benefit of reanalysis depends on the completeness of phenotypic information, the timing of reanalysis, and access to complementary genomic and functional investigations [93]. For cases that remain unresolved after routine testing, cross-institutional data sharing, case matching, multi-omics analyses, and functional validation can provide critical evidence for establishing variant pathogenicity. A recent study combined prenatal genome sequencing with international case aggregation, transcriptomic profiling, and zebrafish functional experiments, thereby identifying biallelic *SNAPIN* variants as the cause of a prenatal-onset neurodevelopmental disorder [96]. Similarly, combining genome sequencing with transcriptome sequencing, long-read sequencing, advanced bioinformatic analyses, and functional assays can further improve diagnostic yield in critically ill children with suspected genetic disorders [97]. Genomic diagnosis should therefore be regarded as a dynamic and iterative process in which clinical observations generate research hypotheses, while research findings are continuously fed back into and translated into clinical practice.

These technical blind spots highlight the need for complementary genomic approaches rather than reliance on a single platform. GS, LRS, and OGM provide complementary rather than interchangeable information. GS enables scalable detection of SNVs, small indels, and CNVs; LRS improves the detection of repeat expansions, haplotype phasing, and nucleotide-level resolution of complex breakpoints; whereas OGM enables chromosome-scale characterization of balanced and complex structural rearrangements [98,99]. In preimplantation genetic testing and in fetuses with complex structural anomalies, combining GS with LRS or OGM can facilitate further characterization of suspected repeat expansions, complex structural variants, and chromosomal rearrangements [100]. Accordingly, a tiered workflow guided by clinical phenotypes and initial testing results may be adopted: short-read GS can serve as a broad initial test, followed by LRS for suspected repeat expansions or difficult genomic regions and OGM for unresolved or suspected complex structural variants. Such technological integration may broaden variant detection and improve diagnostic yield.

From a clinical implementation perspective, molecular diagnostic technologies should be stratified according to the maturity of supporting evidence into routine, indication-specific, and investigational applications. Sequencing-based expanded carrier screening [101], cfDNA screening for common fetal trisomies [62,73,75,77], and CMA for fetuses with structural anomalies [45,46] are established components of routine clinical practice. PGT-M and PGT-SR are clinically appropriate for couples with known familial pathogenic variants or chromosomal structural rearrangements [17,18,19,20,102]. For fetuses with structural anomalies and nondiagnostic CMA results, trio exome sequencing may be considered when a monogenic disorder is suspected, provided that detailed phenotyping, multidisciplinary review, and pre- and post-test genetic counseling are available [103]. In contrast, routine PGT-A for all in vitro fertilization patients has not consistently improved cumulative live-birth outcomes [104]. Non-invasive PGT [2,3,4,5,26,27,105], broad single-gene disorder screening using maternal plasma cfDNA [63,64,65,66,67,68,69,70,71,79], OGM [58], and LRS [12,57] are currently better positioned as indication-specific complementary or investigational approaches rather than universal first-line tests. Their broader adoption is limited by insufficient large-scale clinical validation, embryonic mosaicism or maternal DNA contamination, specialized sample requirements, interpretive complexity, and cost. Among emerging technologies, short-read genome sequencing may be the most likely to consolidate parts of the current sequential CMA-followed-by-ES pathway because it can detect multiple variant classes in a single assay [43,103,106]. However, GS cannot yet replace karyotyping, targeted repeat-expansion assays, targeted methylation testing for suspected imprinting disorders, or other orthogonal methods in all clinical contexts [103]. Cost-effectiveness should therefore be evaluated across the entire diagnostic pathway rather than by test price alone, incorporating diagnostic yield, turnaround time, avoided sequential testing, genetic counseling, confirmatory testing, and downstream clinical management [107,108]. Accordingly, clinical workflows should remain phenotype- and indication-driven: established tests should be used first, followed by ES or GS in unresolved cases, whereas OGM, LRS, or functional assays should be reserved primarily for suspected variant classes or technically challenging genomic regions. Table 4 summarizes the clinical status, major limitations, and expected roles of the principal technologies.

Although non-invasive technologies are advancing rapidly, a cautious approach remains essential. However, it must be clearly recognized that for aneuploidy screening, the positive predictive value of expanded NIPT for microdeletions/microduplications remains relatively low; indiscriminate expansion of the screening scope may lead to unnecessary invasive diagnostics and maternal anxiety. Due to their intrinsic chromosomal instability, trophectoderm cells are prone to aneuploidy [109]. Furthermore, embryonic mosaicism arises during early embryonic development, and the limited number of trophectoderm cells obtained by biopsy may not accurately represent the genetic composition of the inner cell mass [110]. This “TE-ICM (Inner Cell Mass) discordance” can lead to false-positive or false-negative results in preimplantation genetic testing [111]. Concurrently, the limited sample size of TE biopsies often results in whole-genome amplification bias and allelic dropout, which subsequently triggers the misidentification of target loci. Therefore, clinical application should remain prudent to avoid overpromising [105].

As detection technologies advance, the detection rate of Variants of Uncertain Significance (VUS) has risen exponentially. This places a substantial interpretive burden on genetic counselors and may cause considerable anxiety among pregnant women [112]. The ACMG recommends reporting only pathogenic or likely pathogenic variants, with VUS reported only under specific circumstances (e.g., when the partner is a confirmed carrier of a pathogenic variant via screening and only with patient consent) [101]. There is an urgent clinical need to establish standardized reporting protocols for VUS to prevent unnecessary pregnancy terminations due to over-interpretation. Artificial intelligence is actively being applied to resolve VUS interpretation challenges [113]. Prenatal phenotype-driven interpretation of ES and GS remains constrained by the incomplete and continuously evolving representation of fetal abnormalities in the human phenotype ontology (HPO). In 2022, the HPO consortium added 95 prenatal phenotype terms to 152 existing terms and further revised their definitions, synonyms, and disease annotations, indicating that prenatal phenotypic standardization still requires continued refinement [114]. Fetal abnormalities may also emerge or evolve during pregnancy, and additional phenotypic information obtained through serial ultrasound examinations, postnatal assessment, or postmortem examination can refine candidate-gene prioritization and variant interpretation [93,115]. Reanalysis of unresolved prenatal ES cases has shown that updated phenotyping and newly established gene–disease associations may alter molecular interpretation, although the additional diagnostic yield remains modest [93]. Prenatal genomic workflows should therefore incorporate serial phenotyping, updated HPO annotations, multidisciplinary review, reverse phenotyping, and periodic reanalysis [93,115,116].

Integrated analysis of DNA and transcriptomic data is becoming increasingly important in the diagnosis of genetic disorders, with RNA-seq gradually transitioning from a research tool to a clinical adjunct. By detecting aberrant splicing, expression outliers, monoallelic expression, and transcript loss, RNA-seq provides functional evidence complementary to ES/GS. It can therefore facilitate the reclassification of variants of uncertain significance and improve the interpretation of deep-intronic and splice-altering variants in particular [117,118,119]. In postnatal rare-disease cohorts, integrated DNA–RNA analysis has provided additional diagnoses in approximately 12–16% of previously unresolved cases and increased the overall diagnostic rate from 31% to 38% in a heterogeneous cohort [117,118,119]. In prenatal diagnosis, RNA-seq of cultured amniotic-fluid cells has been used to characterize the transcript-level consequences of variants in *CHD7*, *COL1A2*, and *MYRF*, thereby supporting variant reclassification [120]. An integrated diagnostic workflow may therefore begin with ES/GS-based variant discovery, followed by RNA-seq of a clinically relevant tissue for unresolved splice-altering or non-coding variants, joint analysis of aberrant splicing, gene expression, and allele-specific expression, and orthogonal functional validation when required [97,117,118,119,120]. In a rapid rare-disease diagnostic program, the addition of transcriptomic analysis and targeted functional assays increased the diagnostic yield from 47% to 54% [97]. This *SNAPIN* study further exemplifies how transcriptomic and functional evidence can complement prenatal ES/GS to confirm gene–disease relationships and clarify the underlying pathogenic mechanisms [96]. However, tissue-specific gene expression, RNA quality, turnaround time, and the lack of standardized reference datasets remain major barriers to routine clinical implementation. With the experimental application of Polygenic Risk Scores in embryo selection [31], we must be vigilant against sliding into the ethical abyss of “eugenics,” clearly distinguishing the boundary between disease prevention and trait selection.

In summary, the synergistic development of molecular diagnostic methods has contributed to the development of a more integrated framework for genetic disease prevention and reproductive management. The focus of the future should not merely be on pursuing greater sequencing depth, but on achieving the broader implementation of precision prenatal genomic medicine through multi-omics data integration, AI-assisted decision-making, and standardized ethical pathways.

## 5. Conclusions

This review provides an overview of the current landscape of molecular diagnostic technologies across the preconception, preimplantation, and prenatal stages. LRS has improved the detection of variants in complex genomic regions during preconception screening. niPGT should remain classified as an investigational embryo-assessment approach and should not be conflated with celocentesis or other invasive prenatal sampling procedures. Concurrently, AI and deep-learning methods may support maternal DNA deconvolution, embryo assessment, and variant prioritization, but remain investigational because prospective validation and evidence of clinical benefit are limited. However, technical feasibility must not be conflated with clinical utility; expanding the scope of NIPT may lead to unnecessary invasive diagnostics and increased maternal anxiety. Future reproductive medicine may increasingly move beyond the use of isolated diagnostic tools toward more integrated life-course genomic management. Future studies should determine whether emerging transcriptomic and methylomic approaches can achieve sufficient analytical standardization, gestational-age-specific interpretation, and prospective clinical utility before routine implementation.

## Figures and Tables

**Table 1:** Comparison of Clinical Performance Among Major Prenatal Molecular Diagnostic Technologies.

Technology Type	CMA	ES	NIPT
Diagnostic Yield	~4–10% (for pathogenic CNVs [41]	Incremental clinical yield of ~5–12% [41]	PPV ~28–80% [42]
Turnaround Time	~10–12 days [43]	~21–26 days [43]	~7 days [44]
Indications for Use	First-tier test for structural abnormalities.	Used when CMA is negative but multi-system anomalies or monogenic diseases are suspected.	Used solely for prenatal screening; positive results require invasive confirmation.
Primary Targets	Aneuploidies and CNVs.	Point mutations, small insertions/deletions (indels) in exonic regions.	Trisomy 21, 18, and 13; sex chromosome aneuploidies.

Note: CMA, Chromosomal Microarray Analysis; ES, Exome Sequencing; NIPT, Non-invasive Prenatal Testing; PPV, positive predictive value; CNVs, Copy Number Variants.

**Table 2:** Types of Genetic Variants and Corresponding Detection Technologies.

Variant Type	Corresponding Detection Technology
Aneuploidies/Copy Number Variants (CNVs)	CMA is recommended as a first-tier diagnostic test for fetuses with structural anomalies, while karyotyping remains valuable for selected chromosomal abnormalities and balanced rearrangements. Low-pass genome sequencing (LP-GS) demonstrates diagnostic efficacy comparable to CMA for CNVs.
Balanced Structural Variants (SVs)	Karyotyping remains the gold standard for the visual identification of rearrangements. Fluorescence in situ hybridization (FISH) serves for targeted verification and adjunctive diagnosis. OGM, as an emerging high-resolution technology capable of precise breakpoint localization, is transitioning from research to clinical validation.
Single Nucleotide Variants (SNVs)/Indels	Targeted testing is indicated for cases with a clear family history and known pathogenic loci. ES demonstrates superior efficacy for single-gene disorders, particularly in fetuses with multiple structural anomalies and negative CMA results. GS broadens variant coverage but incurs higher costs.
Regions of Homozygosity (ROH)	ROH can be reliably detected by SNP-based chromosomal microarray analysis but not by oligonucleotide-only array comparative genomic hybridization. Large or chromosome-specific ROH may suggest parental consanguinity or uniparental isodisomy; parental studies and targeted molecular confirmation may be required.
Repeat Expansions (REs)	PCR-based methods/Southern blotting remain the gold standard but suffer from low throughput, targeting single disorders. Bioinformatics approaches for short-read sequencing enable multiplex detection but require subsequent validation. OGM and LRS represent cutting-edge technologies capable of directly resolving many repeat expansion regions.
Uniparental Disomy (UPD)	SNP-array can identify whole-chromosome or segmental isodisomy through absence of heterozygosity but may fail to detect pure heterodisomy. Confirmation may require parental SNP/short tandem repeat analysis and, for imprinted regions, targeted methylation testing.
Mosaicism	Karyotyping, SNP-array/CMA, FISH, and sequencing-based methods can detect mosaicism, but sensitivity depends on the affected tissue, abnormal-cell fraction, culture conditions, sequencing depth, and platform-specific thresholds. Low-level or tissue-limited mosaicism may require testing of an alternative sample or targeted confirmatory analysis.

Note: Aneuploidies/Copy Number Variants, numerical chromosomal abnormalities (gain/loss) and deletions/duplications of DNA segments; Balanced Structural Variants, chromosomal rearrangements without alteration in copy number; Single Nucleotide Variants/Indels, intragenic mutations, such as point mutations and frameshifts; Regions of Homozygosity, identity of Single Nucleotide Polymorphisms across homologous chromosomal segments; Repeat Expansions, abnormal expansion of short tandem repeat sequences.

**Table 3:** Comparison of Non-Invasive Prenatal Testing Methods.

Technology	Characteristics	Accuracy Metrics	Detection Scope	Target Population
Non-Invasive Prenatal Testing (NIPT) [42,62,63]	Simple operation; short turnaround time; excellent screening performance for common aneuploidies. However, it is strictly a screening tool requiring invasive validation for positive results; positive predictive value for small-fragment CNVs is low.	Sensitivity ≥ 98.96%, Specificity ≥ 99.94% for Trisomy 21/18/13 and Sex Chromosome Aneuploidies.	Common chromosomal aneuploidies; Expanded version detects CNVs (≥100 kb) and select monogenic disorders.	Pregnancies undergoing aneuploidy screening without fetal structural abnormalities or ultrasound findings strongly suggestive of a genetic disorder.
Relative Haplotype Dosage Analysis (RHDO) [64,65]	Capable of detecting various monogenic diseases with high diagnostic accuracy. Unable to detect de novo fetal variants.	Accuracy in validation cases 100% (*n* = 70); Classification accuracy in optimized consanguineous families 93.9% (*n* = 8).	Monogenic hereditary diseases.	Pregnant women at high risk for monogenic diseases.
COATE-seq [63,66]	Enables “one-stop” detection of aneuploidies, microdeletions/microduplications, and monogenic variants; effectively eliminates interference from maternal CNVs and multiple pregnancies. Technical workflow is complex with high sequencing costs.	High-risk pregnancy population: Sensitivity 98.5%, Specificity 99.3%, positive predictive value (*n* = 133).	Common aneuploidies; classical microdeletion/microduplication regions; common monogenic diseases.	High-risk populations (ultrasound anomalies, high-risk serum screen); Pregnant women requiring simultaneous screening for multiple genetic disorders.
RMD + Targeted Sequencing [67]	No paternal or proband samples required; simultaneous detection of multiple variants with a relatively short turnaround time. Results are unstable at low fetal fractions (<4%).	Clinical sensitivity 100%, Specificity 100% (*n* = 64, results were stable when the fetal fraction was ≥4%).	Monogenic hereditary diseases (sickle cell disease)	Pregnant women at high risk for monogenic diseases.
Nanopore- RHDO [68]	No proband samples required; potential cost-effectiveness. SNP detection accuracy is lower than NGS platforms, requiring correction with NGS data; operation is complex.	Concordance with invasive diagnostic testing: 92.3% (*n* = 13).	Monogenic hereditary disease (β-thalassemia)	Pregnant women at high risk for monogenic diseases.
cSMART [69]	Covers the full coding region of target genes; high genotyping accuracy; suitable for regions with complex mutational spectra.	Overall concordance with invasive diagnosis 96.97%, Sensitivity 100%, Specificity 96.15% (*n* = 33).	Monogenic hereditary diseases (phenylketonuria)	Pregnant women at high risk for monogenic diseases.
Single Circulating Trophoblast (SCT) Testing [70]	Diagnostic-level performance; free from maternal DNA interference; supports fetal status validation after PGT-M. Cells are rare, leading to a failure rate of 18.1%; complex operation; highly dependent on gestational age (≤14 weeks).	Sensitivity and specificity for aneuploidy are both 100% in nine families.	Chromosomal aneuploidies, CNVs, Monogenic diseases, Post-PGT-M prenatal confirmation.	Singleton/Twin pregnancies; High-risk populations (advanced maternal age, high-risk serum screen, family history); Contraindications to invasive prenatal diagnosis.
Genome-wide Haplotype Analysis [71]	Simultaneous detection of monogenic diseases and aneuploidies; high accuracy in haplotype reconstruction; supports fetal status validation after PGT-M. Unable to detect de novo mutations.	The average concordance of fetal haplotype with embryo biopsy/neonate was 97%.	Genome-wide haplotype analysis, Monogenic diseases, Aneuploidies	Families who have undergone PGT-M; Families at high risk for monogenic diseases/aneuploidy.

Note: Pregnant women at high risk for monogenic diseases include those with a family history of monogenic diseases, a reproductive history of children with monogenic diseases, patients with monogenic diseases, or carriers of relevant variants. RHDO, Relative Haplotype Dosage Analysis; COATE-seq, Collaborative Allele-aware Targeted Enrichment Sequencing; RMD, Relative Mutation Dosage; cSMART, Circulating Single-Molecule Amplification and Resequencing Technology.

**Table 4:** Clinical status and future roles of major technologies.

Technology	Current Clinical Status	Major Limitations	Expected Role
NGS-based expanded carrier screening	Routine	Residual risk and incomplete detection of complex variants.	Main preconception screening platform, supplemented by targeted assays or LRS.
PGT-M and PGT-SR	Routine for defined indications	Embryo biopsy, amplification bias, allelic dropout, and counseling requirements.	Established option for couples with known familial variants or chromosomal rearrangements.
PGT-A and niPGT	Indication-specific or investigational	Mosaicism, uncertain outcome benefit, low DNA input, and maternal contamination.	PGT-A may remain selective; niPGT cannot currently replace trophectoderm biopsy.
NIPT for common trisomies	Routine screening	Placental origin, low fetal fraction, and false-positive or false-negative results.	Standard aneuploidy screening, but not a diagnostic test.
Expanded NIPT and monogenic cfDNA testing	Investigational or selected use	Variable PPV, low disease prevalence, and limited prospective validation.	Complementary screening in selected populations; cannot replace invasive diagnosis.
CMA and trio ES	Routine or indication-specific diagnosis	Limited variant coverage, incomplete fetal phenotyping, and Variants of Uncertain Significance (VUS) interpretation.	Current core pathway for fetal structural anomalies.
Short-read GS	Emerging first-tier option	Cost, interpretation burden, incidental findings, and incomplete detection of selected variant classes.	Most likely to consolidate parts of the CMA-followed-by-ES pathway.
LRS, OGM, RNA sequencing (RNA-seq), and other multi-omics approaches	Complementary or investigational	Specialized samples, high cost, limited standardization, and insufficient clinical validation.	Targeted use in unresolved or technically complex cases.

## Data Availability

No new data were created or analyzed in this study. Data sharing is not applicable to this article.

## Supplementary Materials

The following supporting information can be downloaded at: https://www.mdpi.com/article/10.3390/cimb48080756/s1.

## Abbreviations

The following abbreviations are used in this manuscript:

ACMG	American College of Medical Genetics and Genomics
ADO	Allelic Dropout
AI	Artificial Intelligence
BDA	Blocker Displacement Amplification
CAHEA	Comprehensive Analysis of Hemophilia A
CAPKD	Comprehensive Analysis of Polycystic Kidney Disease
CATSA	Comprehensive Analysis of Thalassemia by Single-molecule sequencing
CMA	Chromosomal Microarray Analysis
CNN	Convolutional Neural Network
CNV	Copy Number Variant
CNV-seq	Copy Number Variation Sequencing
COATE-seq	Collaborative Allele-aware Targeted Enrichment Sequencing
CPM	Confined Placental Mosaicism
cSMART	Circulating Single-Molecule Amplification and Resequencing Technology
DECENT	Deep learning model for maternal DNA decontamination
ES	Exome Sequencing
ECS	Expanded Carrier Screening
EVs	Extracellular Vesicles
FF	Fetal Fraction
FISH	Fluorescence In Situ Hybridization
GS	Genome Sequencing
HPO	Human Phenotype Ontology
ICM	Inner Cell Mass
ICSI	Intracytoplasmic Sperm Injection
IVF	In Vitro Fertilization
LRS	Long-Read Sequencing
LSTM	Long Short-Term Memory
MCC	Maternal Cell Contamination
NGS	Next-Generation Sequencing
NIPT	Non-Invasive Prenatal Testing
niPGT	Non-Invasive Preimplantation Genetic Testing
OGM	Optical Genome Mapping
PPV	Positive Predictive Value
PGT	Preimplantation Genetic Testing
PGT-A	Preimplantation Genetic Testing for Aneuploidies
PGT-M	Preimplantation Genetic Testing for Monogenic disorders
PGT-SR	Preimplantation Genetic Testing for Structural Rearrangements
RHDO	Relative Haplotype Dosage Analysis
RMD	Relative Mutation Dosage
RE	Repeat Expansions
ROH	Regions of Homozygosity
RNA-seq	RNA Sequencing
SCT	Single Circulating Trophoblast
SGD	Single-Gene Disease
SECM	Spent Embryo Culture Medium
SMRT	Single-Molecule Real-Time sequencing
SNP	Single Nucleotide Polymorphism
SNV	Single Nucleotide Variant
SVs	Structural Variants
TE	Trophectoderm
UMI	Unique Molecular Identifiers
UPD	Uniparental Disomy
VUS	Variants of Uncertain Significance
