# Supplementary material for: Technological Advances in Molecular Diagnostic Methods for Hereditary Diseases in Preconception and Prenatal Settings
Source: Curr Issues Mol Biol. 2026 Jul 25;48(8):756. doi: 10.3390/cimb48080756 (PMC13511703; doi:10.3390/cimb48080756)
Supplement: Supplementary file 1 [file cimb-48-00756-s001.zip › cimb-4406003-supplementary.pdf]

Table S1. Major professional guidelines and consensus statements included in this review.

Organization	Year	Topic	Main recommendation
ACOG [6]	2017	Carrier screening	Carrier-screening information should be offered to all pregnant individuals and preferably before pregnancy. When one partner is identified as a carrier, testing of the reproductive partner and genetic counseling should be offered.
ACMG [101]	2021	Expanded carrier screening	Recommends a population-neutral, tiered carrier-screening framework and offering Tier 3 screening for autosomal recessive and X-linked conditions to individuals planning pregnancy or already pregnant. Residual risk and reproductive options should be discussed.
ASRM [102]	2023	PGT-M	PGT-M should be considered according to the clinical indication, inheritance pattern, disease severity, and technical feasibility. Individualized test development, nondirective genetic counseling, and discussion of confirmatory prenatal testing are recommended.
ASRM [104]	2024	PGT-A	Current evidence does not support PGT-A as universal routine screening for all IVF patients. Its use should be individualized, with counseling regarding limitations, mosaic results, and uncertain effects on cumulative live-birth outcomes.
ISPD [103]	2022	Prenatal ES and GS	Prenatal genome-wide sequencing may be considered for selected fetuses with structural abnormalities after nondiagnostic standard testing. Trio analysis, standardized laboratory procedures, multidisciplinary interpretation, and pre- and post-test counseling are recommended.

ISPD [75]	2023	NIPT for fetal chromosomal conditions	Supports NIPT as a highly accurate screening approach for common trisomies while emphasizing condition-specific performance, pretest counseling, management of no-call results, and invasive diagnostic confirmation of positive results. Evidence remains insufficient for routine screening of several rare chromosomal conditions.
SMFM [77]	2025	cfDNA screening for aneuploidies	Recommends cfDNA as the most sensitive and specific screening test for trisomies 21, 18, and 13 and that it be made available to all obstetric patients. cfDNA remains a screening rather than diagnostic test; routine population screening for microdeletions or genome-wide CNVs is not recommended.
